# Multilocus phylogeny and cryptic diversity in Asian shrew-like moles (*Uropsilus*, Talpidae): implications for taxonomy and conservation

**DOI:** 10.1186/1471-2148-13-232

**Published:** 2013-10-25

**Authors:** Tao Wan, Kai He, Xue-Long Jiang

**Affiliations:** 1 State Key Laboratory of Genetic Resources and Evolution, Kunming Institute of Zoology, Chinese Academy of Sciences, Kunming 650223, China; 2 University of Chinese Academy of Sciences, Beijing 100049 China; 3 Department of Biological Sciences, University of Manitoba, Winnipeg, MB R3T 2N2 Canada

## Abstract

**Background:**

The genus *Uropsilus* comprises a group of terrestrial, montane mammals endemic to the Hengduan and adjacent mountains. These animals are the most primitive living talpids. The taxonomy has been primarily based on cursory morphological comparisons and the evolutionary affinities are little known. To provide insight into the systematics of this group, we estimated the first multi-locus phylogeny and conducted species delimitation, including taxon sampling throughout their distribution range.

**Results:**

We obtained two mitochondrial genes (~1, 985 bp) and eight nuclear genes (~4, 345 bp) from 56 specimens. Ten distinct evolutionary lineages were recovered from the three recognized species, eight of which were recognized as species/putative species. Five of these putative species were found to be masquerading as the gracile shrew mole. The divergence time estimation results indicated that climate change since the last Miocene and the uplift of the Himalayas may have resulted in the diversification and speciation of *Uropsilus*.

**Conclusions:**

The cryptic diversity found in this study indicated that the number of species is strongly underestimated under the current taxonomy. Two synonyms of *gracilis* (*atronates* and *nivatus*) should be given full species status, and the taxonomic status of another three potential species should be evaluated using extensive taxon sampling, comprehensive morphological, and morphometric approaches. Consequently, the conservation status of *Uropsilus* spp. should also be re-evaluated, as most of the species/potential species have very limited distribution.

## Background

The Asian shrew-like moles (*Uropsilus* spp.) are the only living genus in Uropsilinae and encompass the most primitive subfamily of Talpidae [1,2]. In contrast to semi-fossorial shrew moles, fossorial moles, and aquatic desmans, the members of *Uropsilus* possess a shrew-like body shape, conspicuous external ears, a slender tail as long as the head-body length, and compressed claws without specialized characters for burrowing. All of these characters indicate an ambulatory lifestyle [3]. Although the ancestor species of Uropsilinae were widely distributed throughout Eurasia, the distribution of the relict genus *Uropsilus* is limited to southwest China and north Myanmar, in middle to high montane forests. The basal position of Uropsilinae in the family is supported by morphological characters [4,5] and DNA sequences [2,6,7], although the evolutionary relationships within this genus remain poorly understood. Despite a recent description of a new species (*Uropsilus aequodonenia*) using morphometric and molecular approaches [8], the classifications of living uropsilines has been based exclusively on morphological and morphometric comparisons, and the taxonomy has changed several times (Table 1): three genera (*Nasillus, Rhynchonax*, and *Uropsilus* ), five species (*U*. *aequodonenia*, *N. investigator*, *R. andersoni*, *N. gracilis*, and *U. soricipes*) and two subspecies (*R. a. atronates* and *R. a. nivatus*) have been named (Table 1). Five taxa are currently recognized as full species of the genus *Uropsilus* (*aequodonenia*, *andersoni*, *gracilis*, *investigator*, and *soricipes*), whereas the other two (*atronates* and *nivatus*) are recognized as synonyms of *U. gracilis*[1,9]. However, only a few *CYT B* sequences are available for four of the recognized species, and most of the taxon sampling was limited to western Sichuan [8]; furthermore, there are no available sequences for *U. investigator*. Thus, it would be interesting to examine whether the current taxonomy can be recovered by molecular approaches and whether morphological cryptic species exist within the widely distributed species [10].

**Table 1:** Major Disagreements in the taxonomy of Uropsilinae

**Allen, 1938 **[3]		**Ellerman & Morrison-Scott, 1951 **[11]		**Hoffmann, 1984 **[12]		**Nowak, 1999 **[13]		**Present study**	
		**Cranbrook, 1960 **[14]				**Hutterer, 2005 **[9]			
		**Corbet, 1980 **[15]				**Hoffmann & Lunde, 2008 **[1]			
		**Honacki et al. 1982 **[16]							
**Genus**	**Species** **(subspecies)**	**Genus**	**Species** **(subspecies)**	**Genus**	**Species** **(subspecies)**	**Genus**	**Species** **(subspecies)**	**Genus**	**Species** **(subspecies)**
*Uropsilus*	*soricipes*	*Uropsilus*	*soricipes*	*Uropsilus*	*soricipes*	*Uropsilus*	*soricipes*	*Uropsilus*	*aequodonenia*^*d*^
*Rhynchonax*	*andersoni*		*(soricipes)*		*gracilis*^*b*^		*gracilis*^*c*^		*andersoni*
	*(atronates)*		*(gracilis)*		*andersoni*		*andersoni*		*atronates*
	*(nivatus)*		*(andersoni*^*a*^*)*				*investigator*		*gracilis*
*Nasillus*	*gracilis*		*(investigator)*						*investigator*
	*investigator*		*(nivatus)*						*nivatus*
									*soricipes*
									sp. 1
									sp. 2
									sp. 3

^*a*^*atronates* is included as synonym.

^*b*^*atronates*, *nivatus*, and *investigator* are included as synonyms.

^*c*^*atronates* and *nivatus* are included as synonyms.

^*d*^ recognized by Liu et al. [8].

The mountains of southwest China and adjacent areas harbor an extremely high biodiversity [17]. Two nonexclusive hypotheses, including a pre-Pleistocene diversification model and a Pleistocene refugia model have been proposed to explain the expansion of endemic species [18]. Under the pre-Pleistocene diversification model, the uplifting of the Qinghai-Tibetan Plateau (QTP) in association with climate changes boosted allopatric speciation (e.g., [19-21]). Conversely, Quaternary glacial-interglacial cycles caused repeated shifts in distribution ranges [22], which may also have motivated diversification and speciation in isolated mountain chains [23-25]. Given that Uropsilinae might have evolved for approx. 30 million years [26], this genus could be a promising model to test these two hypotheses.

In this study, we sampled Asian shrew-like moles throughout their distribution range, sequenced both mitochondrial and nuclear loci, and adopted molecular phylogenetic and species delimitation approaches to represent a systematic framework of the genus *Uropsilus*. Our goals are the following: (i) to examine the proposed taxonomy based on morphology/morphometrics; (ii) to assess the evolutionary relationships among living taxa; and (iii) to test alternative scenarios of species/diversification patterns within the genus.

## Results

### Sequence characteristics

We obtained 6,330 bp sequences for each voucher specimen, including 1,985 bp mitochondrial (*CYT B* [1,140 bp] and 12S rRNA [845 bp]) and 4,345 bp nuclear (*ADORA3* [321 bp], *ATP7A* [675 bp], *BDNF* [555 bp], *BMI1* [313 bp], *CREM* [389 bp], *PLCB4* [331 bp], *RAG1* [1,010 bp], and *RAG2* [751 bp]) sequences. A total of 552 sequences were deposited in GenBank with Accession nos. from KF777818 to KF778377 (Additional file 1: Table S1). No premature stop codon was observed in the coding regions of the protein coding genes examined. The mitochondrial genes showed relatively higher genetic polymorphisms than the nuclear genes (Additional file 2: Table S2). Note that the *BMI1* gene had no variable site and was thus not used in the species delimitation or network tree reconstruction.

### Gene trees and divergence times

**Figure 1:**
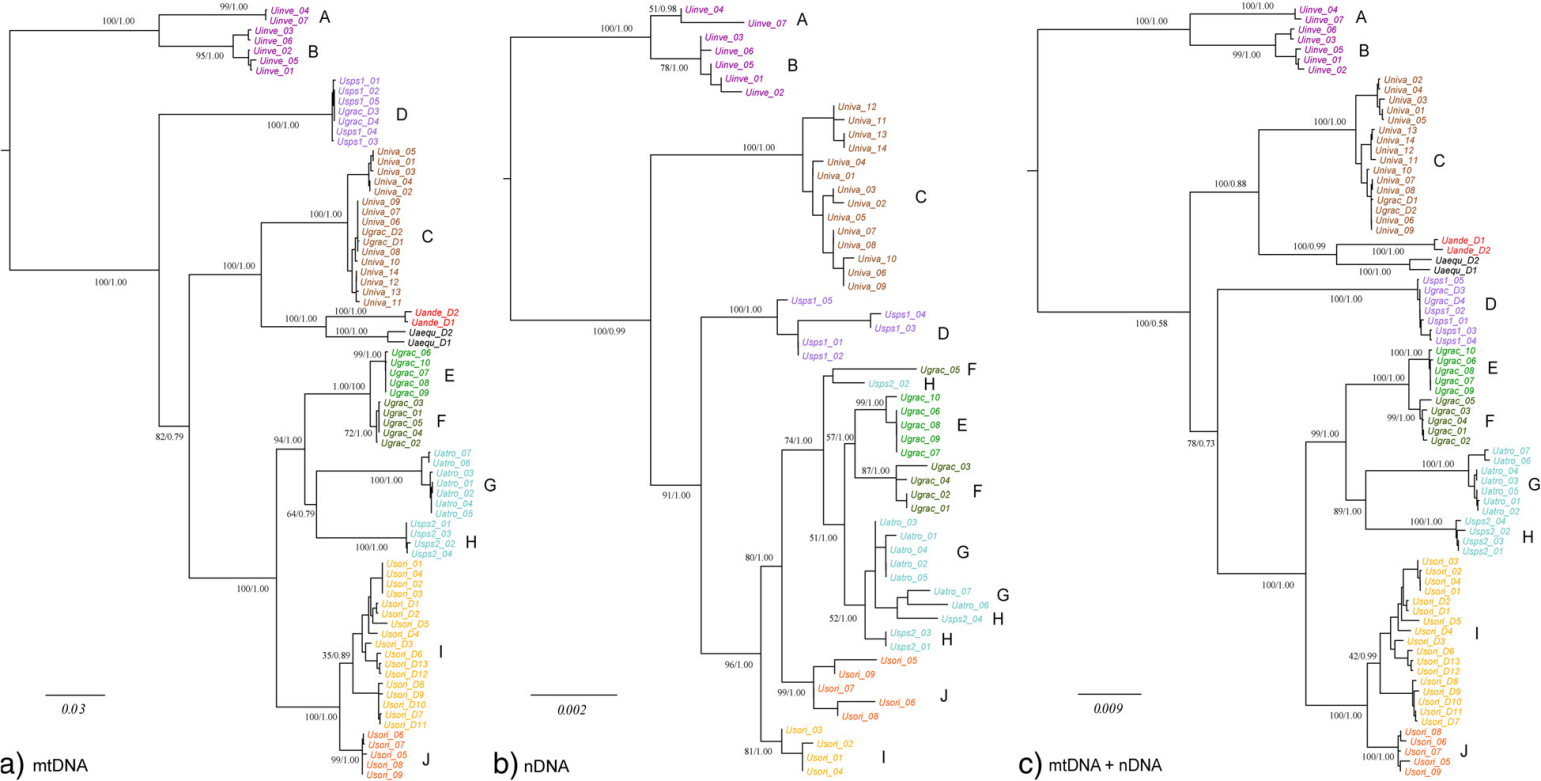
**Concatenated gene trees of genus *Uropsilus *based on Bayesian and ML analyses.** ML gene trees of *Uropsilus* based on: **(a)** two mitochondrial genes, **(b)** eight nuclear genes, and **(c)** a combined mitochondrial-nuclear dataset. The node numbers represent the maximum-likelihood bootstraps [BS] and Bayesian posterior probabilities [PP]. A-J represents clade derived from the mitochondrial tree estimation.

The Bayesian and Maximum likelihood (ML) phylogenetic reconstructions recovered very similar topologies; only the ML gene trees are shown (Figure 1). In addition to *U. aequodonenia* and *U. andersoni*, 10 distinct lineages were recovered in the mitochondrial gene tree (i.e., clades A-J; Figure 1a), and the monophyly of all these lineages was strongly supported (i.e., maximum-likelihood bootstrap proportions [BS] ≥ 85, Bayesian posterior probability [PP] ≥ 0.95). Two of these clades represented *U. investigator* (A and B), two represented *U. soricipes* (I and J), and the others represented *U. gracilis* (C-H). The relationships among the clades were overall strongly supported, except at two nodes. *U. aequodonenia* and *U. andersoni* were supported as sister species (BS = 100, PP = 1.0) and are sister to clade C (BS = 100, PP = 1.0). *U. gracilis* was supported as polyphyletic. The nuclear gene tree differed from the mitochondrial gene tree in not recovering the monophyly of clades E, F, and G, even though E + F + G + H was still supported as monophyletic (BS = 74, PP = 1.0; Figure 1b). Moreover, clade J was not supported as the sister group of clade I, but is sister to clade E + F + G + H + I (BS = 96, PP = 1.0). The mitochondrial-nuclear combined gene tree was very similar to the mitochondrial gene tree (Figure 1c) but supported clade C as the sister taxon of the other clades, except clade A + B (BS = 100, PP = 0.58). The partitioned branch support scores showed that both the mitochondrial and nuclear datasets support all but two relationships each (PBS < 1; Additional file 3: Figure S1).

**Figure 2:**
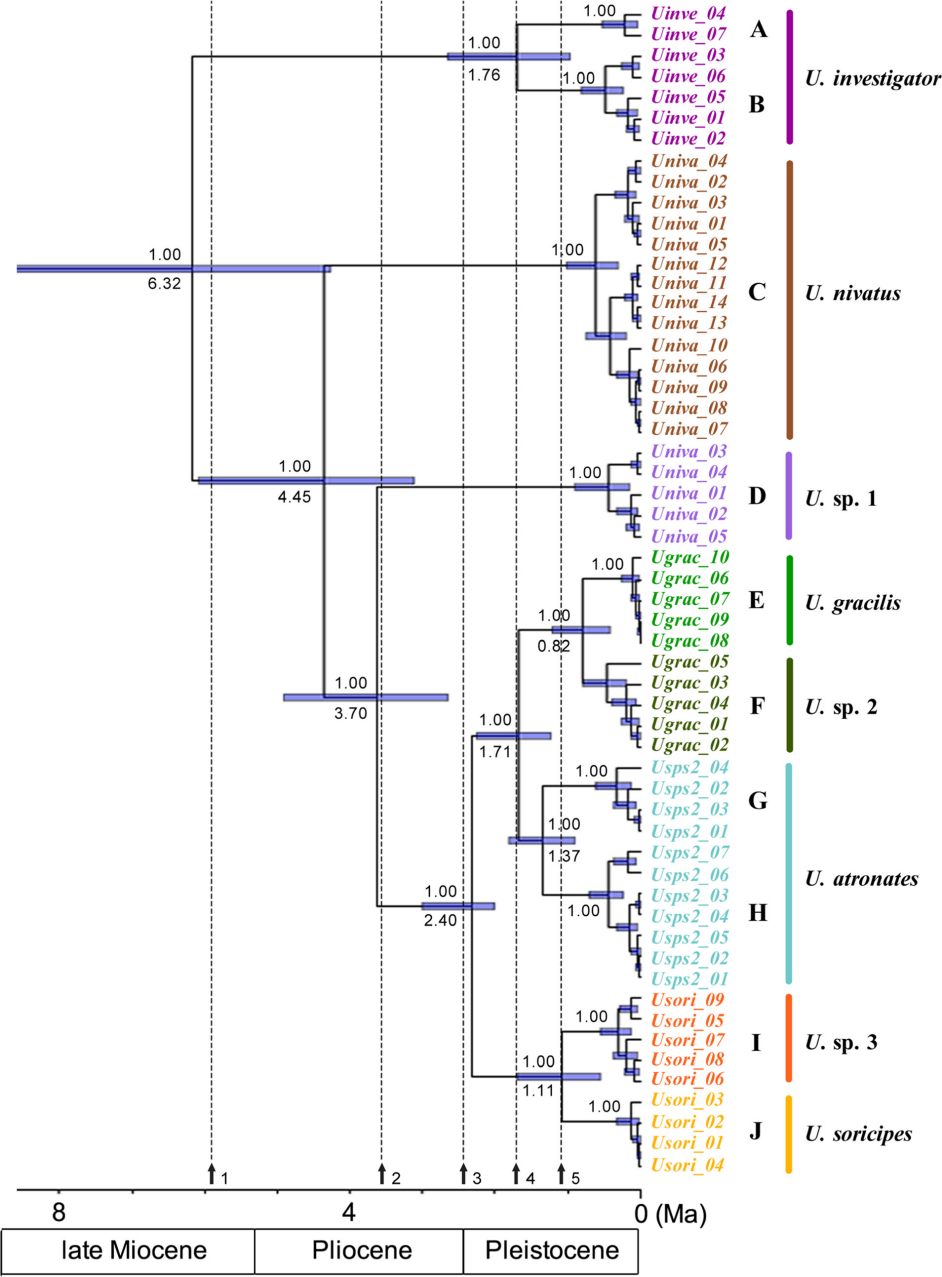
**Maximum credibility ultrametric tree of *Uropsilus *based on mitochondrial-nuclear concatenated data.** The numbers above branches are the Bayesian posterior probabilities and the numbers under the branches represent the median divergence times. The node bars indicate the 95% interval confidence for the divergence time estimates. The five vertical lines represent the major historical events in the diversification of *Uropsilus*: 1) 5.93 Ma, Messinian salinity crisis [27]; 2) 3.6 Ma, strongest uplift of the QTP [28,29]; 3) 2.4 Ma, modern Asian monsoon system began and Pleistocene glaciation started [30,31]; 4) 1.8 Ma, uplift of the QTP [32]; 5) 1.1 Ma, uplift of QTP [28,32].

The ultrametric tree derived from the divergence time estimation is shown in Figure 2. It is noteworthy that all relationships were strongly supported. The most recent common ancestor of *Uropsilus* occurred in the latest Miocene (6.18 Ma, 95% CI: 8.65-4.27), and the following divergences occurred from 4.35 Ma to 1.37 Ma (6.08-0.91).

### Species delimitation and species trees

**Figure 3:**
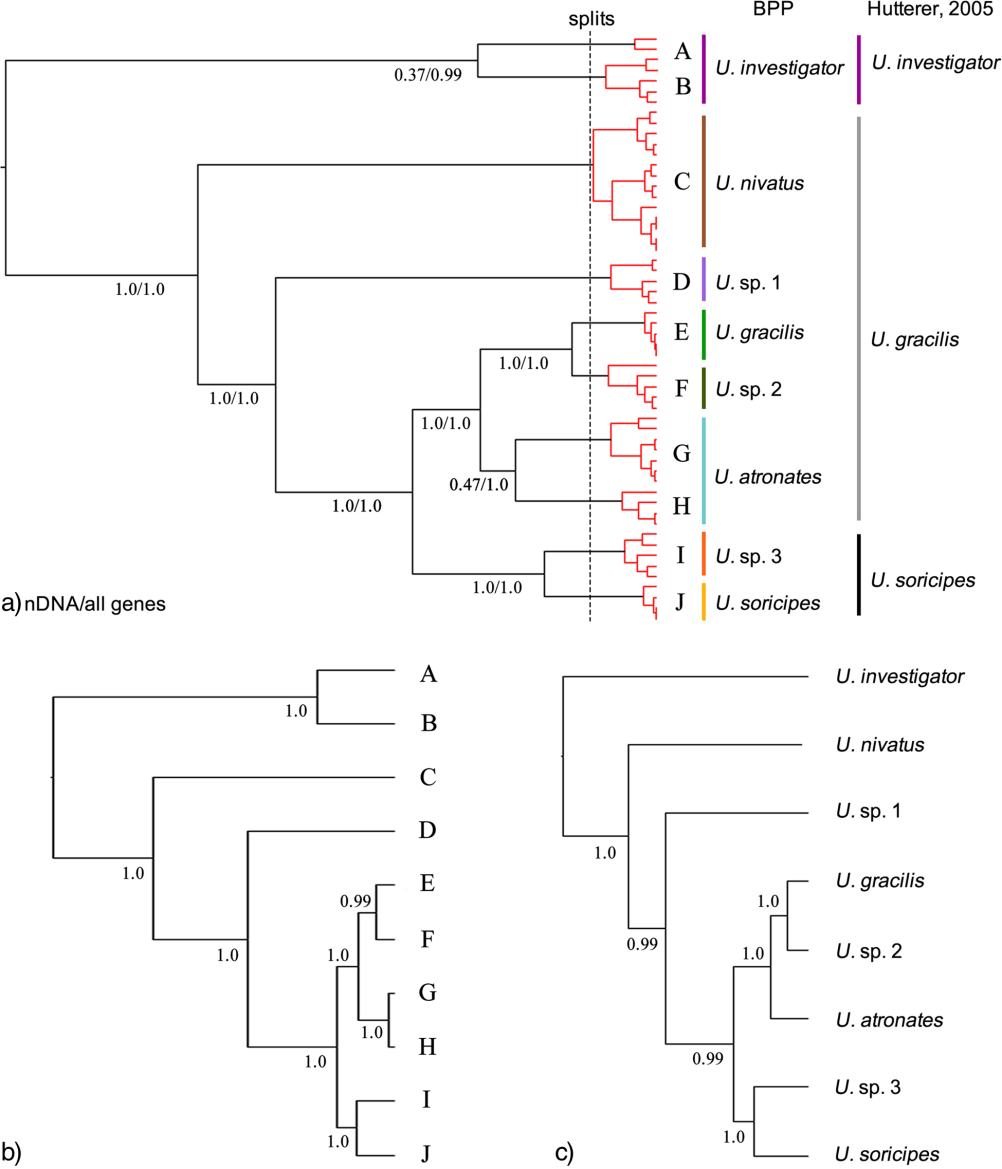
Results of species delimitation using splits and BPP, and species trees reconstructed using the *BEAST model. Node numbers indicate Bayesian posterior probabilities supporting each clade as two putative species when using nuclear genes alone and using all genes, respectively **(a)**; and Bayesian posterior probabilities estimated in *BEAST analyses **(b and c)**.

**Figure 4:**
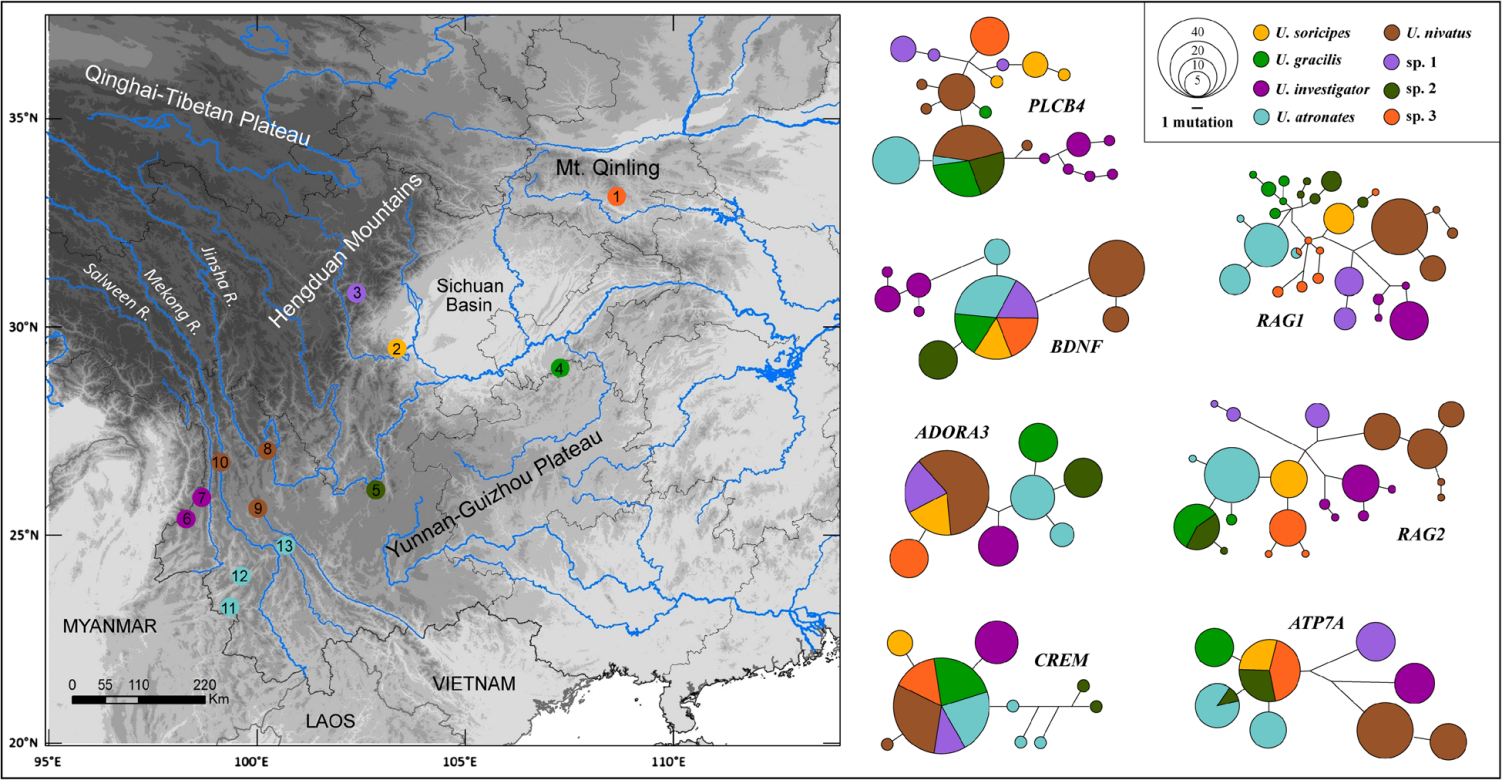
Nuclear gene network trees and distribution of each putative species.

The split recognized clades diverged older than 0.61 Ma as putative species; therefore, all 10 monophyletic clades in the ML tree were recognized as putative species (Figure 3a). The K2P distance between clades E and F was calculated to be 0.013 and 0.039 between I and J. All of the other pairwise distances between the clades are ranged from 0.085 to 0.203 (Additional file 4: Table S4). The *BEAST analysis recovered the same topology as the mitochondrial-nuclear concatenated gene tree (Figure 3b), which was used as the guide species tree for the BPP analyses. In each Bayesian delimitation analysis, the posteriors of all parameters had a high ESS (> 1000); the results are provided in Additional file 5: Table S4. When using both mitochondrial and nuclear genes, BPP recognized all 10 clades as putative species (PP ≥ 0.99; Figure 3a; Additional file 5: Table S4). However, when using nuclear genes alone, the posterior supports for clades A and B and, I and J as distinct species were much lower (PP ≤ 0.47; Figure 3a; Additional file 5: Table S4). Following a conservative approach, we recognized A + B and G + H as single species each. We again reconstructed a species tree using the 8-species scenario, and the topology did not change (Figure 3c). In addition, the 7 nuclear gene network trees showed that *U. investigator* did not share any haplotypes with other species/putative species when the other 7 species/putative species shared haplotypes in 3 to 4 genes (Figure 4).

## Discussion

### Mitochondrial vs. nuclear genes

Although incongruences between mitochondrial and nuclear or between different nuclear genes have been observed in many studies (e.g., [33]), the mitochondrial gene trees and PBS analyses suggested that in our case mitochondrial genes still represent good genetic markers. When both mitochondrial and nuclear genes alone failed to fully resolve the relationships, combined mitochondrial and nuclear genes using both concatenated and coalescent approaches recovered identical topologies and were robustly supported. It follows that a combination of both rapidly evolving mitochondrial and conservative nuclear genes appears to be the best approach to resolve ancient but closely spaced divergences.

### Species delimitation and taxonomic reappraisal

The accuracy of molecular species delimitation has been discussed several times [34,35]. BPP has been recognized as the most accurate approach when compared to other coalescent-based methods like ABC and SpeDeSTEM [34]. Recently, Miralles and Vences found that the number of putative species strikingly ranged from 9 to 34, depending on the method implemented [35]. Although BPP was not recognized as the best approach in their study, the results could be affected by small sample size of each putative species and the small number of loci (see [36] for detail). Despite the uncertainty of the number of putative species recognized, molecular data represent only a crude estimate rather than a final conclusion and should be diagnosed using comprehensive morphological characters.

Splits and BPP recognized 10 and 8 putative species, respectively. It is of note that clades A + B and G + H were recognized as one species each in the BPP analyses, even the pairwise K2P distances of *CYT B* were higher than 0.100. This value is higher than the distance between sister species *U. aequodonenia* and *U. andersoni* (0.085) and the average genetic distance between sister species of mammals (0.081; [37]). This result may be due to the small sample size of clade A (n = 2) and potential gene flow (see [36]) because A and B are sympatrically distributed and G and H were not supported as monophyletic by nuclear genes (Figure 1b). On the other hand, *U.* sp. 2, *U*. sp. 3 were recognized as putative species even though the K2P distance between them and their sister taxa were very low (K2P = 0.013-0.039), which might due to mitochondrial introgression or different lineage sorting scenarios. We treated *U.* spp. 2 and 3 as putative species here, as the BPP analyses suggested there was no recent gene flow. Regardless of the ambiguity of species delimitation, the high pairwise genetic distances and the polyphyly of previously known *U. gracilis* strongly suggested that number of species of *Uropsilus* has been underestimated.

With respect to the previous taxonomy of *Uropsilus*, dental formulas have been used as a key for species diagnosis. Four dental formulas have been observed, and *U. gracilis* and *U. investigator* share the same dentition (Additional file 3: Figure S1; [12]). Our results revealed that six polyphyletic taxa (species/putative species) share the same dental formula (i2/1,c1/1,p4/4,m3/3 = 38; Additional file 3: Figure S1). Within the six species/putative species, clade E was sampled from the type locality of *U. gracilis*, and can be safely assigned to this species (Figure 4). The type locality of *U. investigator* is distributed to the west of the Mekong River, geographically close to the clades A and B, thus we assigned clade A + B to *U. investigator*. Clades G + H and C consist of populations from the type localities of *atronates* and *nivatus*, respectively, and these two taxa should most likely be recognized as valid species. There is no available name for spp. 1–3, and their taxonomic status should be evaluated based on extensive sampling and comprehensive morphological/morphometric comparisons.

### Divergence pattern of *Uropsilus*

The results of our divergence time estimation suggested that neither the pre-Pleistocene speciation nor the Pleistocene “speciation pump” hypothesis could exclusively explain the speciation/diversification pattern. Indeed, the diversification of *Uropsilus* may have been affected by periodical orogenic processes and climate change. Prevailing trends toward cooling and desiccation in the late Miocene (i.e., Messinian salinity crisis; [27,38]) and the Pliocene/Pleistocene boundary [39] have been well documented, and leading to the diversification of humid-dwelling taxa (e.g., [40,41]). Therefore, global cooling may be responsible for the splits of the Asian shrew-like moles at 6.18 Ma and 2.40 Ma. Similarly, geological studies have supported rapid uplifts of QTP at 3.6 Ma, 1.8 and 1.1 Ma [28,30], which may also have resulted in the diversifications of the genus.

The distribution of different species/putative species showed a strong geographic pattern, which could be partly due to the extremely complex topography, understory habitats, and low dispersal ability of the animals [42,43]. Nonetheless, at least four species/putative species, including *U. aequodonenia*, *U. andersoni*, *U. soricipes*, and *U.* sp.1, are distributed in the western Sichuan mountains, indicating a very complex geographic history. Extensive sampling is required to uncover the vicariant, migration, and speciation history patterns.

### Cryptic diversity and conservation implications

Identifying cryptic diversity is essential for the accurate assessment of genetic diversity and conservation planning [10,44]. Cryptic divergence and strong geographic structures have been observed in other endemic taxa [45,46], indicating that topography has strong effect on diversification, particularly with regard to small and sedentary animals. The current conservation statuses of *Uropsilus* spp. are all considered as “Least Concern” or “Data Deficient” (IUCN Red List Category; [47]), due to their presumed wide distribution and/or large population size [48]. However, our results indicated that unrecognized species exist within *Uropsilus*, and most of the species/putative species have very limited distribution. Indeed, because of global warming and continuous habitat loss, the species diversity of the genus may be actually threatened. Therefore, re-evaluation of the endangered categories relying on a new systematic background is warranted; at present, these putative species should be considered as evolutionary significant units and taken into consideration for conservation planning.

## Conclusions

In the present study, we obtained sequences of *Uropsilus* throughout their distribution in the mountains of southwest China. We reconstructed a robust phylogeny for this most primitive talpid genus and found cryptic diversity. Five putative species were determined in addition to the five recognized species. We suggested that *atronates* and *nivatus* should be recognized as full species, and a comprehensive morphological diagnosis is warranted for three unidentified species. Moreover, the conservation statues should re-evaluated, as most of the species/putative have limited distribution. Finally, the divergence of the genus may be affected by climatic changes and tectonic activities, providing clues for the expansion of endemic fauna.

## Methods

### Sample collection and ethics statement

**Figure 5:**
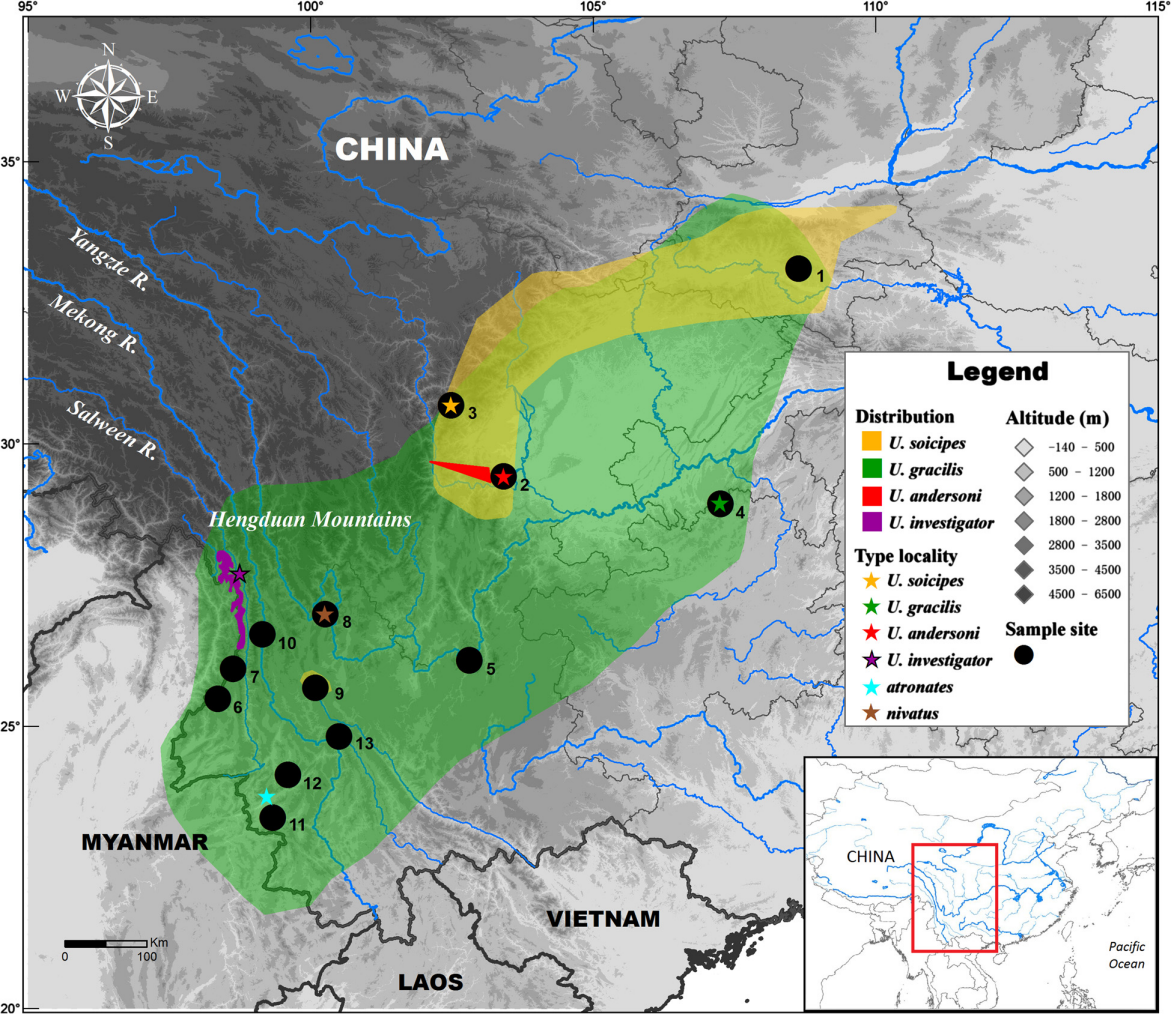
**Distribution, type locality, and sampling sites of *Uropsilus *spp.** The coloured ranges indicate for the natural distribution of four *Uropsilus* species. Species distribution maps are modified from the the IUCN Red List of Threatened Species. Version 2013.1. (http://www.iucnredlist.org). Downloaded on 09 June 2013.

A total of 56 individuals were collected, representing 3 of the 5 named species from 13 localities throughout the distribution area. These localities include the type localities of the *gracilis* (Mt. Jinfo, Chongqing), *soricipes* (Baoxing, Sichuan), synonyms *atronates* (Mucheng, Yunnan), and *nivatus* (Mt. Yulong, Yunnan) species (Figure 5, Table 2). All of the animal samples were obtained following the regulations of China for the implementation of the protection of terrestrial wild animals (State Council Decree [1992] No. 13) and approved by the Ethics Committee of Kunming Institute of Zoology, Chinese Academy of Sciences, China (no specific permit number). Voucher specimens were deposited in the Kunming Institute of Zoology, Chinese Academy of Sciences, Kunming, China.

**Table 2:** Samples used in this study

**No.**	**Locality**	**Coordinates**	**Clade (s)**	**Original identification**	**Putative species**
1	Mt. Qinling, Shannxi	N33.5°, E108.5°	I	*U. soricipes*	sp. 3
2	Mt. Emei, Sichuan	N29.3°, E103.5°	J	*U. soricipes*	*U. soricipes*
3	Baoxing, Sichuan	N30.7°, E102.7°	D	*U. gracilis*	sp. 1
4	Mt. Jinfo, Chongqing	N29.0°, E107.2°	E	*U. gracilis*	*U. gracilis*
5	Dongchuan, Yunnan	N26.0°, E103.0°	F	*U. gracilis*	sp. 2
6	Tengchong, Yunnan	N25.5°, E98.2°	A, B	*U. investigator*	*U. investigator*
7	Mt. Gaoligong, Yunnan	N26.0°, E98.7°	B	*U. investigator*	*U. investigator*
8	Mt. Yulong, Yunnan	N27.3°, E100.2°	C	*U. gracilis*	*U. nivatus*
9	Mt. Diancang, Yunnan	N25.7°, E100.1°	C	*U. gracilis*	*U. nivatus*
10	Mt. Biluo, Yunnan	N26.6°, E99.0°	C	*U. gracilis*	*U. nivatus*
11	Gengma, Yunnan	N23.5°, E99.2°	G	*U. gracilis*	*U. atronates*
12	Mt. Yongde, Yunnan	N24.1°, E99.6°	G	*U. gracilis*	*U. atronates*
13	Mt. Wuliang, Yunnan	N24.4°, E100.7°	H	*U. gracilis*	*U. atronates*

Numbers refer to the locality numbers presented in Figure 5 and 4. The putative species names are based on their distribution, the type locality of each species and the results of the BPP species delimitation (see Discussion).

### Laboratory protocols

All samples were frozen in ethanol at−70°C prior to DNA extraction. Total genomic DNA was extracted by a standard phenol/chloroform method [49] or using the DNeasy Tissue kit (Qiagen, Gemamy) from either liver or muscle tissues. Two mitochondrial (*CYT B*, 12S rRNA) and eight nuclear (adenosine A3 receptor [*ADORA3*], *ATP7A*, brain-derived neurotrophic factor [*BDNF*], polycomb ring finger oncogene [*BMI1*], cAMP responsive element modulator [*CREM*], phospholipase C beta 4 [*PLCB4*], recombination activating protein 1 [*RAG1*], and recombination activating protein 1 [*RAG2*]), genes including the coding sequences (CDSs) and 3′ untranslated regions (3′ UTRs) were applied. All these genes have been widely used in mammalian phylogenetic studies, including the mammal Tree of Life project (e.g., [50]). The gene fragments were amplified with rtaq DNA Polymerase (TaKaRa, China). Touchdown PCR was used to increase the specificity, sensitivity and yield [51]. The primers and high and low annealing temperatures are provided in Table 3. All PCR products were purified using the UNIQ-10 spin column DNA gel extraction kit (Sangon, China). Sequencing was performed using the BigDye Terminator Cycle kit v3.1 (Invitrogen, USA) and an ABI 3730xl sequencer (Applied Biosystems, USA).

**Table 3:** Primers used for PCR and sequencing

***Locus***	**Primer name**	**Primer sequences (5′-3′)s**	**Sense/anti-sense**	Annealing temperature (°C) *	**Citation**
**12S**	L613_hk1	GGCGGGCGAGCAAAGCACTGAAAATG	Sense	53-48	[40]
	H1478_hk1	TGATTGGTGGAGGGTGACGAGCGGTGTGT	Anti-sense		
***CYT B***	L14724_hk3	GGACTTATGACATGAAAAATCATCGTTG	Sense	60-55	[40]
	H15915_hk3	GATTCCCCATTTCTGGTTTACAAGAC	Anti-sense		
***RAG1***	F1705	GCTTTGATGGACATGGAAGAAGACAT	Sense	53-48	[52]
	R2951	GAGCCATCCCTCTCAATAATTTCAGG	Anti-sense		
***RAG2***	RAG2-F220	GATTCCTGCTAYCTYCCTCCTCT	Sense	58-53	[52]
	RAG2-R995	CCCATGTTGCTTCCAAACCATA	Anti-sense		
***ADORA3***	ADORA3-F	ACCCCCATGTTTGGCTGGAA	Sense	60-55	[50]
	ADORA3-R	GATAGGGTTCATCATGGAGTT	Anti-sense		
***ATP7A***	ATP7A-F	TCCCTGGACAATCAAGAAGC	Sense	60-55	[50]
	ATP7A-R	AAGGTAGCATCAAATCCCATGT	Anti-sense		
***BDNF***	BDNF-F	CATCCTTTTCCTTACTATGGTT	Sense	60-55	[50]
	BDNF-R	TTCCAGTGCCTTTTGTCTATG	Anti-sense		
***BMI1***	BMI1-F	CATTGGGCCATAGTTTGTTAATCTCAA	Sense	60-55	[50]
	BMI1-R	CCAATATGGCATTGTACAACAAGC	Anti-sense		
***CREM***	CREM-F	AGGAACTCAAGGCCCTCAAA	Sense	60-55	[50]
	CREM-R	GGGAGGACAAATGTCTTTCAA	Anti-sense		
***PLCB4***	PLCB4-F	GTGAAATTGGAAGCCGAGAT	Sense	63-60	[50]
	PLCB4-R	CACCAAGCTCATTTACTTGTGA	Anti-sense		

* Touchdown PCR programs were used for the amplification of each gene fragment. The higher annealing temperature was used in the first cycle and decreased by 0.5°C per cycles in the first 10 cycle. The lower annealing temperature was used for the next 25 cycles.

### Sequence assembling and alignment

Sequences were assembled and edited using DNASTAR Lasergene version 7.1. All genes were aligned in MUSCLE [53] and further examined by eye in MEGA5 [54]. In addition, the *CYT B* sequences determined in a recent study representing *Uropsilus aequodonenia*, *U. andersoni*, *U. gracilis* and *U*. *soricipes* were downloaded from GenBank [8]*.* Sequences of *Talpa altaica* of the subfamily Talpinae and *Sorex araneus* of the family Soricidae were chosen as outgroup taxa (Additional file 1: Table S1).

### Phylogenetic analyses and divergence time estimation

To reconstruct the phylogenetic relationships of *Uropsilus*, ML analyses were performed using RAxML v7.3.2 [55], and Bayesian inference (BI) was performed using BEAST v1.7.5 [56]. The phylogenetic analyses were conducted on the following four datasets: 1) a mitochondrial combined gene dataset; 2) a nuclear gene combined dataset; 3) an all gene combined dataset; 4) the same as dataset 3 but with the *CYT B* sequences downloaded from GenBank removed (Additional file 6). We used a partitioning strategy to incorporate the variation in evolutionary processes among different sites [57]. The best-fit partitioning scheme and the appropriate model of DNA evolution for each partition were determined in PartitionFinder v1.0 [58]. The alignment was partitioned by gene and by codon position. Twelve models of molecular evolution (K80, HKY, TrNef, TrN, SYM, GTR, K80 + G, HKY + G, TrNef + G, TrN + G, SYM + G, GTR + G) were compared and ranked by the Bayesian Information Criterion (BIC) [59]. The best partitioning scheme and substitution models are given in Additional file 7. The ML analyses were performed using the CIPRES Science Gateway [60]. We selected the GTR + gamma model for each partition and the rapid Bootstrapping algorithm (Stamatakis A, Hoover P, Rougemont J: A Rapid Bootstrap Algorithm for the RAxML, Web-Servers, unpublished) and ran 500 bootstrap replicates. The Bayesian phylogenetic trees were calculated in BEAST v1.7.5 [56]. We employed relaxed uncorrelated exponential clock models that allowed the rate in each branch to evolve independently [61]. The combined mitochondrial fragment and each nuclear gene were given specific exponential clock models, and a Continuous-time Markov chains (CTMCs) model was employed as a prior for each clock model [62]. Each analysis used a random staring tree, a birth-death tree prior and the program’s default prior distribution of the model parameters. Each analysis was run for 50 million generations with a sampling interval of 5,000 was conducted. Trace v1.5 [63] was used to confirm the effective sample sizes (ESSs) as greater than 200 and the first 30% of the generations were treated as burn-in. The BEAST analyses were repeated four times. To assess the support for the mitochondrial and nuclear genes at each node, partitioned branch support scores (PBSs; [64]) were calculated using TreeRot v2.0 [65] and PAUP 4.0b10 [66]. We performed this analysis using dataset 4. We further constructed network trees for each nuclear gene using NETWORK v4.611; the nuclear genes were unphased with DnaSP v5.10 [67] using the algorithms provided by PHASE [68], and the unphased haplotypes were used to construct median-joining haplotype networks [69]. We ran the MP calculation post-processing option to delete all of the superfluous median vectors and chose one of the shortest trees.

The molecular divergence time was estimated using BEAST v1.7.5. Because mitochondrial genes evolve much faster than nuclear genes [70], missed nuclear genes will lead to inaccurate estimates of branch lengths and divergence times. Therefore, we used dataset 4 for the molecular dating analyses with *U. aequodonenia* and *U. andersoni* excluded. For the fossil-calibrated age constraints, lognormal and exponential distributions were used to account for uncertainty in fossil calibrations [71]. Three calibrations were used. (i) The split of the most recent common ancestor (MRCA) shared by Talpidae and Erinaceidae + Soricidae was at approximately 73 (78–68) million years ago (Ma) [72]. We set up the prior using a lognormal distribution (offset = 63, mean = 10.4, standard deviation = 0.24), such that the mean age was at 73 Ma and the 95% upper boundary was at 78 Ma. (ii) The oldest known Talpini was from the early Oligocene (Palaeogene mammal unit MP 21) [73] at approximately 33.9-32.6 Ma [74]; thus, we set up the prior using an exponential distribution (offset = 32.6, mean = 10.8; 32.6*0.333) [75]. (iii) We employed the oldest known *U. soricipes* from 2.4-2.0 million-year-old strata (early Pleistocene) in Hubei Province, China, and set up the prior using an exponential distribution, (offset = 2.0, mean = 0.67 2.0*0.333) [76,77].

### Species delimitation and species tree estimation

We conducted species delimitation analyses using the “splits” v1.0-14 package (SPecies LImits by Threshold Statistics) for the R statistical environment and the programe BPP v2.2. When using splits, the time-calibrated concatenated gene tree was used as the input tree, whereas the number of putative species was identified using a generalized mixed Yule-coalescent model (GMYC) [78]. We further used BPP v2.2 to conduct a Bayesian species delimitation [79,80]. This software provides the most accurate molecular species delimitation to date [34], and the mixing of the Markov chain is improved in the new version v2.2 [81]. The computational simulation demonstrated that the correct species models could be inferred with high posterior probabilities when 5 or 10 sequences from each population were sampled [36]. A previous study found that a mis-specified guide tree can result in strong support for more species [82]. Therefore, we estimated the species tree using the *BEAST model implemented in BEAST (see below), and assigned the 56 specimens to 10 putative species based on the results of splits (see Results). The analyses were performed using both the mitochondrial-nuclear combined data (dataset 4) and the nuclear genes alone (dataset 2). Gamma prior G (6, 6,000) was used on the population size parameters (θ_s_), and the age of the root in the species tree (τ_0_) was assigned gamma prior G (4, 1,000). Multiple runs were performed using both the species delimitation algorithm 0 and algorithm 1. “Locusrate = 1” specifying the random-rates model of Burgess and Yang [83], or “Heredity = 1” allowing θ to vary among loci, were also used but not at the same time. The analyses for each data set were repeated 12 times. Each rjMCMC was run for 1 million generations and sampled every 10 generations after discarding 10,000 generations as pre-burn-in.

The species trees were estimated using the *BEAST model [84] in BEAST v1.7.5. Because the *BEAST model uses very different assumptions from a concatenated gene tree estimation, we did not use the partitioning scheme derived from Partitionfinder. Instead, we gave 12S rRNA, each codon position of *CYT B* gene and each nuclear gene a different substitution model. The best-fit models were calculated using jModeltest [85] and are provided in Additional file 7. Because *BEAST requires that all species have at least one sequence at each locus, we used dataset 4 for the *BEAST analyses. The 56 specimens were assigned to 8 or 10 putative species based on the results of splits and BPP (see Results). We used the same priors as described above. Each analysis was run for 100 million generations and sampled every 10,000 generations.

### Data accessibility

DNA sequences: accession numbers are provided in Additional file 1: Table S1.

## Competing interests

The authors declare that they have no competing interests.

## Authors’ contributions

TW and KH contributed to the laboratory work, data analyses, and manuscript writing; XLJ conceived the study and commented on the manuscript; all authors have read and approved the final manuscript.

## Supplementary Material

Additional file 1: Table S1 GenBank accession numbers of sequences used in this study. Click here for file

Additional file 2: Table S2 Sequence information of ingroup taxa. Click here for file

Additional file 3: Figure S1 Partition branch support. Click here for file

Additional file 4: Table S3 **Mean** ***CYT B*** **distance between clades calculated using the Kimura 2-parameter.** Click here for file

Additional file 5: Table S4 Results of the BPP analyses based on both mitochondrial-nuclear combined data set and nuclear genes alone. Click here for file

Additional file 6 Datasets 1–4. Click here for file

Additional file 7 Partitioning schemes and molecular evolution model used in the gene tree and species tree estimations. Click here for file
