# Supplementary figures and images for: Multilocus phylogeny and cryptic diversity in Asian shrew-like moles (Uropsilus, Talpidae): implications for taxonomy and conservation
Source: BMC Evol Biol. 2013 Oct 25;13:232. doi: 10.1186/1471-2148-13-232 (PMC3819745; doi:10.1186/1471-2148-13-232)

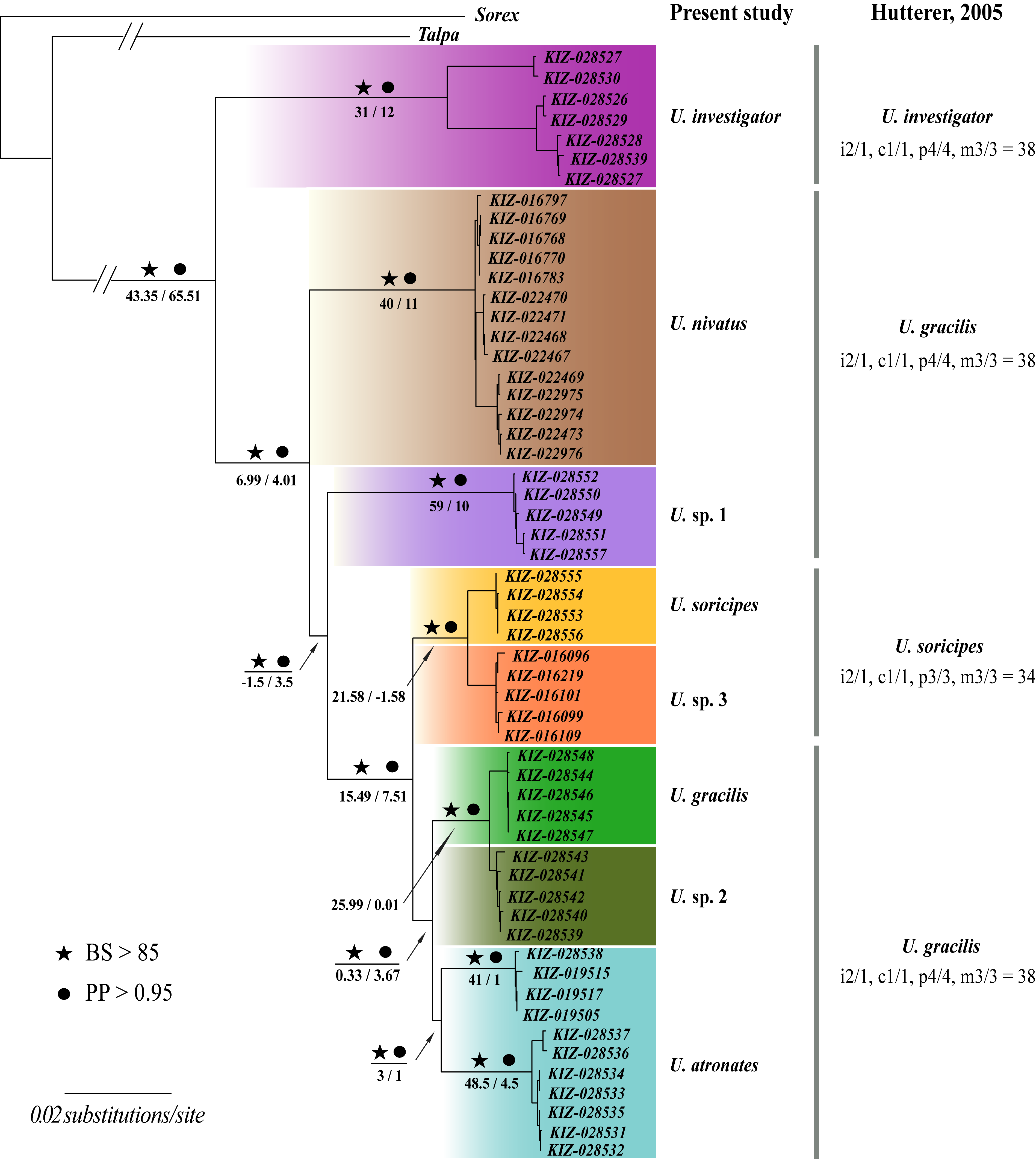

Supplement: Additional file 3: Figure S1 — Partition branch support. [file 1471-2148-13-232-S3.png]
