# Supplementary material for: Multilocus phylogeny and cryptic diversity in Asian shrew-like moles (Uropsilus, Talpidae): implications for taxonomy and conservation
Source: BMC Evol Biol. 2013 Oct 25;13:232. doi: 10.1186/1471-2148-13-232 (PMC3819745; doi:10.1186/1471-2148-13-232)
Supplement: Additional file 7 — Partitioning schemes and molecular evolution model used in the gene tree and species tree estimations. [file 1471-2148-13-232-S7.doc]

**Table S5 - Partitioning schemes and molecular evolution model used in mitochondrial gene tree estimations (data set 1)**

| Subset | Partitions | Best Model |
| --- | --- | --- |
| 1 | 12S | TrN+G |
| 2 | *CYT B*_1st | SYM+G |
| 3 | *CYT B*_2nd | HKY+G |
| 4 | *CYT B*_3 | TrN+G |

**Table S6 - Partitioning schemes and molecular evolution model used in concatenated nuclear gene tree estimations (data set 2**)

| Subset | Partitions | Best Model |
| --- | --- | --- |
| 1 | *ATP7A*_1st, *BDNF*_1st, *BDNF*_2nd, *RAG1*_1st, *RAG2*_1st | HKY+G |
| 2 | *ADORA3_*2nd, *BMI1*, *RAG1_*2nd, *RAG2_*2nd | HKY |
| 3 | *ADORA3_*3rd, *BDNF_*3rd, *RAG1_*3rd | HKY+G |
| 4 | *ATP7A_*3rd, *RAG2_*3rd | HKY |
| 5 | *ADORA3_*1st, *AT_*2nd, *CREM*, *PLCB4* | TrN+G |

**Table S7 - Partitioning schemes and molecular evolution model used in mitochondrial-nuclear concatenated gene tree estimations (data sets 3 and 4)**

| Subset | Partitions | Best Model |
| --- | --- | --- |
| 1 | *ATP7A*_3rd, 12S, *PLCB4* | HKY+G |
| 2 | *CYT B*_1st | TrNef+G |
| 3 | *CYT B*_2nd, *RAG1*_2nd, *RAG2*_2nd, *RAG2*_3rd, *ADORA3*_1st,  *ADORA3_*2nd, *ATP7A_*2nd, *BDNF_*2nd, *CREM*, *PLCB4* | TrN+G |
| 4 | *CYT B*_3rd | HKY+G |
| 5 | *RAG1*_1st, *RAG2*_1st, *ATP7A*_1st, *BDNF*_1st | HKY+G |
| 6 | *RAG1*_3rd, *ADORA3*_3rd, *BDNF*_3rd | HKY+G |

**Table S8 - Best substitution models used in species tree reconstructions**

| Partitions | Best Model |
| --- | --- |
| 12S | GTR+G |
| *CYT B*_1st | SYM+G |
| *CYT B*_2nd | HKY |
| *CYT B*_3rd | HKY+G |
| *RAG1* | K80+G |
| *RAG2* | HKY |
| *ADORA3* | K80 |
| *ATP7A* | HKY |
| *BDNF* | HKY+G |
| *CREM* | HKY |
| *PLCB4* | HKY+G |
